# *Arion vulgaris* pedal epithelium displays fluorescence patterns consistent with uptake of&nbsp; *Nicotiana tabacum* extracellular dye-labeled vesicles *ex vivo*

**DOI:** 10.17912/micropub.biology.001782

**Published:** 2025-12-09

**Authors:** Michaela Liegertová, Monika Iljučoková, Jiří Smejkal, Jan Malý, Michaela Kocholatá

**Affiliations:** 1 Centre for Nanomaterials and Biotechnology (CENAB), Faculty of Science, Jan Evangelista Purkyně University in Ústí nad Labem, Pasteurova 3544/1, 400 96 Ústí nad Labem, Czech Republic; 2 Faculty of Science, Jan Evangelista Purkyně University in Ústí nad Labem, Pasteurova 3544/1, 400 96 Ústí nad Labem

## Abstract

Cross-kingdom trafficking of extracellular vesicles (EVs) is documented between plants and fungal pathogens, yet evidence for plant-herbivore exchange remains scarce. Here we show that EVs isolated from *Nicotiana tabacum* suspension cultures show fluorescence consistent with uptake by the pedal epithelial cells of the herbivorous slug *Arion vulgaris* within 30 min ex vivo. Fluorescently labeled EVs appeared as discrete intracellular puncta enriched in glandular ridges; unlabeled EVs and final-wash controls showed no detectable fluorescence under identical imaging settings. These observations suggest that plant EVs can cross the mucus barrier of a molluscan herbivore, enabling plant-to-slug molecular communication.

**Figure 1. Fluorescence from a DiOC₆(3)‑labeled Nicotiana tabacum extracellular‑vesicle (EV) preparation in the pedal epithelium of Arion vulgaris ex vivo:**
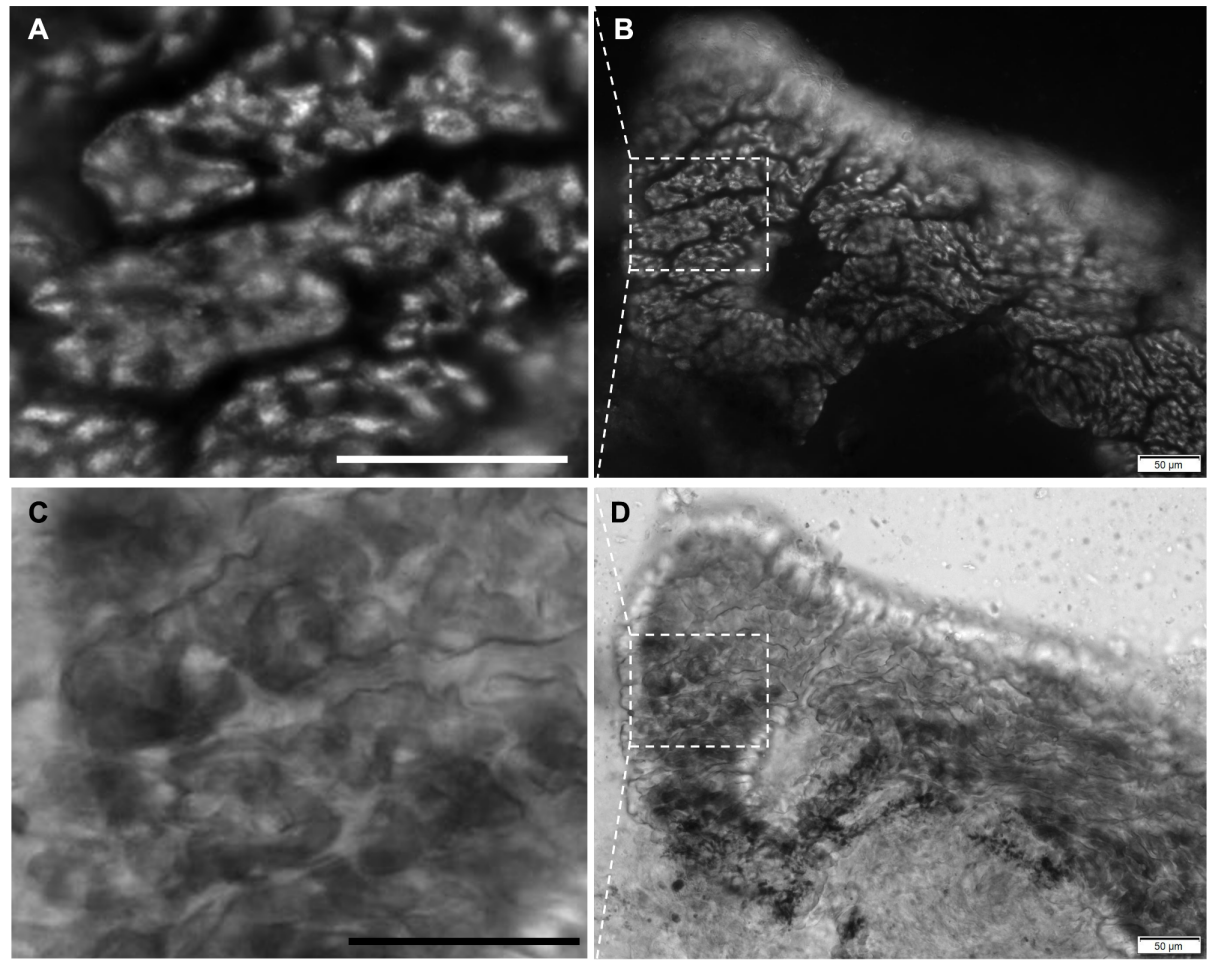
(A) Digital enlargement of the boxed region in (B) showing punctate‑like fluorescence within the apical/supranuclear cytoplasm of columnar epithelial cells. (B) Wide‑field epifluorescence micrograph of a folded pedal tissue fragment after 30 min incubation with the DiOC₆(3)‑labeled EV preparation. (C) Bright‑field image corresponding to (A), outlining the columnar epithelium and underlying glandular structures. (D) Bright‑field overview corresponding to (B); the dashed box indicates the region enlarged in (C). All images were acquired with a 10× objective on a wide‑field epifluorescence microscope; panels A and C are digital enlargements (cropped and resized) of the boxed regions in B and D, respectively. Scale bars: 50 µm. The fluorescence pattern is interpreted as consistent with EV uptake but, given known caveats of lipophilic dyes, does not by itself exclude dye exchange.

## Description

Extracellular vesicles (EVs) are membrane-bound nanoparticles that transfer bioactive molecules, including proteins and RNAs, across cells and species. Cross-kingdom EV exchange is established in plant-pathogen systems, where plants deliver small RNAs via EVs to silence fungal virulence genes (Cai et al., 2018). However, EV-mediated communication between plants and herbivorous animals is largely unknown. The Spanish slug *Arion vulgaris* , an invasive agricultural pest, feeds on plants like tobacco ( *Nicotiana tabacum* ) and secretes mucus rich in EVs that can enter plant cells (Liegertová et al., 2022). To investigate reciprocity, we tested whether tobacco-derived EVs are internalized by slug tissues.

EVs isolated from *N. tabacum* BY-2 suspension culture were labeled with the lipophilic dye DiOC₆(3) and incubated with freshly dissected pedal tissue from *A. vulgaris* . The tissue remained viable, exhibiting contractile movements upon incubation. Fluorescence microscopy revealed bright, punctate-like signals within the supranuclear cytoplasm of epithelial cells ( Figure 1A, B). This apically biased distribution, together with the absence of signal in unlabeled EVs and dye‑depleted supernatant controls, argues against diffuse membrane partitioning and is consistent with early endocytic localization. The fluorescence was restricted to the epithelial layer, with stronger accumulation in glandular ridges and papillae. The corresponding bright-field image ( Figure 1C, D) shows the folded epithelial architecture, with tall columnar cells and abundant glands; the fluorescence pattern aligns with glandular regions of high membrane trafficking activity. The apical bias of puncta suggests early-stage endocytosis, prior to trafficking to perinuclear compartments.

Controls incubated with unlabeled EVs or EV-depleted dye supernatant showed no detectable fluorescence under identical imaging conditions, confirming specificity to labeled EV uptake and ruling out autofluorescence or free dye artifacts. This punctate, intracellular distribution is characteristic of endocytic vesicle trafficking, as seen in other EV uptake systems (Mulcahy et al., 2014).

While these findings are promising, several important limitations warrant consideration. Because lipophilic dyes can form micelles/aggregates and can exchange between membranes, dye‑mediated fluorescence transfer cannot be entirely excluded and orthogonal confirmation with, e.g., GFP‑tagged plant EVs and higher‑NA/confocal imaging will be an important next step.

Next, our experiments were conducted *ex vivo* on excised slug tissue. Although the pedal tissue remained viable and exhibited contractile movements during incubation, this approach cannot fully replicate the physiological conditions of a living slug actively feeding on plant material. *In vivo* factors such as active circulation, metabolic processes, immune responses, and continuous mucus secretion could significantly influence EV uptake dynamics. Demonstrating plant EV internalization *in vivo* by applying labeled EVs to live slug tissue represents a critical next step for establishing physiological relevance.

Lastly, the fate of internalized tobacco EVs and their cargo (proteins, small RNAs, metabolites) within slug cells is unknown. Without characterizing the EV contents and assessing their biological activity, we cannot determine whether this uptake mechanism has tangible effects on slug physiology, gene expression, or behavior. Future functional assays will be essential to establish whether plant-derived molecular cargo can modulate herbivore biology through this pathway.

Our observations were limited to a single time point (30 minutes) and EV concentration (~10¹¹ particles/mL). The kinetics of uptake, dose-response relationships, and long-term fate of internalized vesicles remain uncharacterized. Time-course studies and dose-response experiments would reveal whether EVs traffic deeper into tissues, persist stably, or undergo degradation, providing crucial insights into the efficiency and biological relevance of this communication pathway.

The generalizability of our findings requires further investigation. We focused on one plant source (tobacco cell culture) and one herbivore species ( *A. vulgaris* ). Different plant species may produce EVs with varying compositions affecting uptake efficiency. The mucus-rich pedal epithelium of *A. vulgaris* may be particularly suited for vesicle uptake, a feature not necessarily shared across other herbivore taxa.

To our knowledge, this is the first attempt to demonstrate plant EV internalization by an invertebrate herbivore's cells. Such cross-kingdom transfer could enable plants to deliver defensive cargo (e.g., small RNAs or metabolites) directly into herbivore tissues, potentially influencing slug physiology or behavior. The pedal epithelium, which contacts plants during feeding and locomotion, may serve as a primary entry site. Future studies should characterize EV cargo and functional outcomes to elucidate the role of this pathway in plant-herbivore co-evolution.

## Methods

EV Isolation and Characterization

Nicotiana tabacum Bright Yellow-2 (BY-2) suspension cultures were maintained in Murashige-Skoog medium at 26°C in the dark with shaking at 105 rpm, and subcultured weekly. EVs were isolated from 7-day-old BY-2 culture supernatant by differential ultracentrifugation. The supernatant was first centrifuged at 2000 × g for 20 min at 4°C to remove cells, followed by 10,000 × g for 30 min at 4°C (repeated twice) to remove debris, and then 100,000 × g for 70 min at 4°C (repeated twice) to pellet EVs. The pellet was resuspended in phosphate-buffered saline (PBS) and, to remove large clusters, centrifuged at 5,000 × g for 5 min at 4°C. The final supernatant containing EVs was stored at -20°C. EVs were characterized by nanoparticle tracking analysis using NanoSight NS300 (Malvern Panalytical), with a 562 nm laser, confirming a size range of 50–150 nm consistent with exosomes, following MISEV2018 guidelines (Théry et al., 2018).

EV Fluorescent Labeling

EVs were labeled with DiOC₆(3) (3,3'-dihexyloxacarbocyanine iodide). A 10 mM stock in ethanol was diluted to 100 µM in PBS, incubated with EVs at 37°C for 10 min, and washed three times by ultracentrifugation (100,000 × *g* , 70 min) to remove unbound dye. Supernatant fluorescence confirmed minimal free dye.

Slug Tissue Uptake Assay

Adult *Arion vulgaris* slugs (7–10 cm) were collected locally and maintained in terraria on lettuce. Slugs were euthanized by decapitation. Pedal tissue pieces (~5 × 5 mm) from the foot margin were excised immediately (tissue exhibited contractile movements, indicating viability), rinsed in PBS and incubated in 100 µL labeled EV suspension (~1 × 10¹¹ particles/mL), unlabeled EVs, or dye-depleted supernatant (the final wash supernatant, serving as a negative control for free dye artifacts) at room temperature for 30 min with gentle agitation every 5 min. Tissues were washed in PBS (3 × 5 min) and mounted in PBS for imaging.

Microscopy

Images were acquired on an Olympus IX71 inverted epifluorescence microscope (excitation: 450–490 nm; emission: ~520 nm) using 4x and 10x objectives. Bright-field and fluorescence images were captured with consistent exposure settings.

## Reagents

**Table:** 

Reagent	Source	Identifier
*Nicotiana tabacum* BY-2 cells	Institute of Experimental Botany, Czech Academy of Sciences	N/A
Adult *Arion vulgaris*	Local collection, Czech Republic	N/A
DiOC₆(3)	Invitrogen	Cat# **D273**
ROTI®Fair PBS 7.4	ROTH	Cat# 1108.1
Murashige-Skoog medium	Prepared fresh: 4.3 g/L MS basal salt, supplemented with 0.2 g/L KH₂PO₄, 30 g/L saccharose, 0.1 g/L inositol, 1 mg/L thiamine, 0.2 mg/L 2,4-dichlorophenoxyacetic acid; pH 5.6–5.8, autoclaved at 121 °C for 20 min	N/A
